# Ultrasound in diagnose & treatment follow-up of abdominal pain due to intestinal tuberculosis

**DOI:** 10.1186/2036-7902-7-S1-A5

**Published:** 2015-03-09

**Authors:** MG Ametembun

**Affiliations:** Internal Medicine Dept. and Ultrasound Lab, St Elisabeth Hospital, Flores, Indonesia

**Keywords:** Tuberculosis, Tuberculosis Treatment, Pain Area, Lumen Narrowing, Nodular Structure

## Background

Intestinal tuberculosis frequently causes abdominal pain.

## Objective

To describe ultrasound in management of intestinal tuberculosis.

## Patients and methods

This retrospective study was conducted at St Elisabeth Hospital, Flores, during February 2012 - August 2013 using ALOKA SD1100. Data were abstracted from medical records of all completed treated of intestinal tuberculosis cases.

## Result

N 546(14-85y, x36,5y), 312(57%) female, 234(43%) male.

### Preceding treatment

All with recurrent colic abdomen pain (some very severe), diarrhea/obstipation, distention, “doughy & dam-board phenomena”, constitutional symptoms, cough and X-ray suggestive lung tuberculosis. Intestinal ultrasound on the tympanic area were normal, but on dullness pain area were: a/hypo-peristaltic, irregular thickening heterogenic hypo-echoic, irregular margin of the wall, loss differentiation of the wall layers, with several oval/round nodular structures (patchy hyper-echoic non-shadowing with an irregular rim of lower echo-density, those suggestive granuloma process) within the wall and narrowing of the lumen.

## During 9-12 months of anti tuberculosis treatment

all the symptoms & signs, dullness area, wall thickening, lumen narrowing were disappeared gradually with better peristaltic at the affected area.

### After the treatment

no abdominal pain/tenderness/doughy abdomen/dullness area/ constitutional sign &symptoms anymore. Only 292 (53%) then carried ultrasound at the end of treatment (economic reason) showed intestine wall thickening were decreased and normal peristaltic were seemed on the affected area, but the wall margin were still irregular.

## Conclusion

Ultrasound was very useful for more thorough diagnosis & follow-up the treatment of intestinal tuberculosis.
